# Upadacitinib as a potential management option for diffuse cutaneous systemic sclerosis: A case report

**DOI:** 10.1177/2050313X251343300

**Published:** 2025-06-12

**Authors:** Sidra Sarfaraz, Janis Chang, Mark G. Kirchhof

**Affiliations:** 1 Division of Dermatology, Department of Medicine, The Ottawa Hospital, Ottawa, ON, Canada

**Keywords:** Janus kinase inhibitors, systemic sclerosis, scleroderma, upadacitinib

## Abstract

Systemic sclerosis (SSc) is a complex disease involving vasculopathy, immune dysfunction, and fibrosis, with varied clinical presentations that complicate treatment standardization. It often affects multiple organs, including the skin, lungs, gastrointestinal tract, and kidneys. We present a 52-year-old woman with a 14-year history of diffuse cutaneous SSc with severe, treatment-resistant manifestations. She had Raynaud’s disease with digital ulceration and auto-amputation, telangiectasias, sclerodactyly, esophageal scleroderma, interstitial lung disease, and extensive calcinosis requiring multiple surgeries. Her disease remained poorly controlled despite treatment with nintedanib, sevelamer, colchicine, tadalafil, and prior immunosuppressants such as prednisone and mycophenolate mofetil. We initiated a trial of upadacitinib which resulted in improved vascular and cutaneous symptoms. Overall, upadacitinib provided meaningful clinical benefits despite her refractory, multisystem disease.

## Introduction

Systemic sclerosis (SSc) is a disease characterized by a triad of vasculopathy, immune system dysfunction and fibrosis.^1^ The pathogenesis of SSc remains to be fully elucidated but involves an interplay of genetic and environ-mental factors.^1,2^ Several factors, such as infection and ischemia-reperfusion injuries, cause endothelial cell damage linked to capillary rarefaction and vasculopathy.^2^ Additionally, both innate and adaptive immune systems, as well chemokines and cytokines, are thought to play a role in creating a profibrotic and proinflammatory environment.^1,2^ As a result, there is fibroblast recruitment, activation and proliferation, leading to excess collagen and extracellular matrix deposition in several organs.^1,3^

SSc has a broad spectrum of clinical presentations, making it challenging to create standardized therapeutic guidelines.^4^ The disease frequently features multisystem involvement and may affect organs including the skin, lungs, gastrointestinal tract, or kidneys. Treatment is generally multimodal and targeted to address these varying disease manifestations.^5^ Mycophenolate mofetil is the first-line treatment for skin fibrosis.^5^ Newer, targeted therapies are also being investigated. Nintedanib, a tyrosine kinase inhibitor, was recently approved as the first targeted therapy for SSc-associated interstitial lung disease (ILD).^5^ Additio-nal emerging treatments include anti-interleukin (IL)-6, anti-transforming growth factor (TGF)-β, anti-cytotoxic T-lymphocyte associated (CTLA) protein 4, and Janus-kinase inhibitors (JAKi).^5^ We report a case of a patient with diffuse, multisystem SSc who responded well to upadacitinib, a JAKi.

## Case report

A 52-year-old female was referred to Dermatology for management of extensive, treatment-refractory cutaneous manifestations of SSc. The patient had a 14-year history of diffuse cutaneous SSc, Scl-70 positive, for which she was also followed by Rheumatology and Respirology. Disease manifestations included Raynaud’s disease with digital ulceration and auto-amputation, telangiectasias, sclerodactyly, esophageal scleroderma, ILD, and extensive calcinosis for which she had undergone multiple surgeries. Her disease was poorly controlled despite treatment with nintedanib, sevelamer, colchicine, and tadalafil, as well as previous courses of prednisone. She also failed mycophenolate mofetil, which was discontinued following a postoperative infection from excision of her calcinosis cutis.

**Figure 1.**
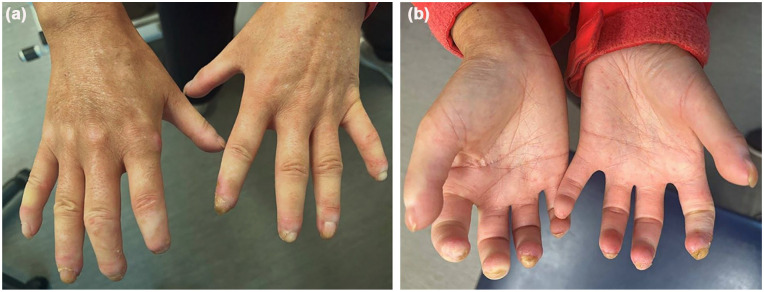
Physical examination of the hands revealed nail dystrophy, digital ulcers, sclerodactyly, and sclerosis, (a) after 2 months of treatment with upadacitinib and (b) 2 months after stopping upadacitinib.

On physical examination, cutaneous findings included facial telangiectasias, microstomia, nail dystrophy, digital ulcers, sclerodactyly limiting range of motion, and sclerosis extending up the forearms (Figure 1). There were also large subcutaneous nodules on the left buttock and right lower back, with CT imaging confirming calcinosis. The largest calcification measured 4.4 × 4.7 × 6.9 cm on CT. Several off-label treatment options were discussed with the patient, including roflumilast, dupilumab, JAKi, and stem cell transplant.

The patient first elected to trial roflumilast, which was ineffective. Her insurance company declined coverage for dupilumab. Therefore, upadacitinib 15 mg daily was initiated. She was treated for 2 months, then lost access to the medication for 1 month due to insurance issues and noted a significant disease flare. Treatment was restarted, with improvement noted based on both patient report and clinical exam. However, she stopped 2 months later due to a transient elevation in aspartate aminotransferase (AST), which later self-resolved and was attributed to a viral infection. She flared again upon holding the upadacitinib, with worsening sclerosis, pain in her digits, and rash on the face and arms. At time of publication, she had not yet restarted the medication due to concerns regarding potential delayed post-operative wound healing.

## Discussion

JAKi are immunomodulators that target one or more enzymes in the JAK family, thereby affecting the JAK/signal transducer and activator of transcription (STAT) pathway.^4^ The JAK/STAT pathway is involved in fibrosis, inflammation, and angiogenesis through cytokines including IL-4, 10, 13, 6, and interferon α, β, and γ.^4^ It is therefore postulated that the anti-inflammatory and antifibrotic properties of JAKi may be beneficial for SSc management.^5^

Of the JAKi, tofacitinib and baricitinib have been trialed in SSc patients with some effect. A recent systematic review assessed the efficacy of these medications in patients with diffuse and limited SSc.^6^ 53% of the patients also had ILD. 88% of patients had cutaneous improvement, with a median reduction of 10 points on the modified Rodnan skin score scale. In addition, 97% of patients had stable or improved ILD, indicated by forced vital capacity volumes. A cutaneous response was noted more often in the patients who had not had previous immunosuppressive therapy compared to patients who were refractory to first-line therapies. Nonetheless, 79% of patients with refractory disease had a cutaneous response.^6^ Side effects of JAKi in SSc patients were uncommon and included minor infections (upper respiratory, urinary tract, and other viral infections), and elevated liver enzymes.^6^

Upadacitinib has not been reported previously as a treatment option for SSc.^6-8^ Our patient tolerated upadacitinib well, with no major adverse events. While she did discontinue the medication due to an isolated elevated AST, this self-resolved and was attributed to a preceding viral infection rather than upadacitinib. Upadacitinib was overall beneficial for our patient’s vascular and cutaneous symptoms, despite the patient’s long-standing history of multisystem, refractory disease.

JAKi, including upadacitinib, shows promise as a potential therapeutic option for SSc patients with treatment-refractory disease. Further research is needed to determine the efficacy of JAKi in treating noncutaneous manifestations of SSc, as well as their impact on morbidity and mortality. Appropriate patient selection and optimal timing of JAKi therapy, with respect to early versus later disease, also needs to be clarified.^7^
